# Highly effective removal of nickel ions from wastewater by calcium-iron layered double hydroxide

**DOI:** 10.3389/fchem.2022.1089690

**Published:** 2023-01-04

**Authors:** Ning Li, Mingjie Yuan, Sheng Lu, Xiaoli Xiong, Zhigang Xie, Yongsheng Liu, Wei Guan

**Affiliations:** ^1^ Department of Chemistry and Chemical Engineering, College of Environment and Resource, Chongqing Technology and Business University, Chongqing, China; ^2^ Department of Artificial Intelligence, Chongqing Technology and Business University, Chongqing, China; ^3^ Chongqing Key Laboratory of Environmental Materials and Remediation Technologies, Chongqing University of Arts and Sciences, Chongqing, China

**Keywords:** heavy metal, calcium-iron layered double hydroxide, nickel ions, isomorphic transition, mechanism

## Abstract

Water pollution due to heavy metals has become a universal environmental problem. Ni(II) is a common heavy metal ion in polluted wastewater, which has high toxicity and carcinogenicity. In this study, the structure of a calcium-iron layered double hydroxide (Ca-Fe-LDHs) was synthesized and characterized by FTIR, XRD, SEM and XPS. Then, Ni(II) ion was effectively removed by Ca-Fe-LDHs and its mechanism for this materials was described. The maximum adsorption capacity of Ni(II) for Ca-Fe-LDHs was 418.9 mg‧g^−1^ when the initial concentration of Ni(II) was 1 g/L. The adsorption and removal of Ni(II) by Ca-Fe-LDHs was attributed to the action of hydroxyl groups on the hydrotalcite, generating surface capture. Ni(OH)_2_)0.75(H_2_O)0.16(NiCO_3_)0.09, Ni(OH)_2_, NiO, NiSO_4_ and other precipitates were generated on its surface. And a small amount of Ni-Fe-LDHs was generated through isomorphic transition before hydrolysis. Therefore, surface capture and isomorphic transition enhanced the removal efficiency of Ni(II) with Ca-Fe-LDHs, making Ca-Fe-LDHs as a potential material for effective removal of Ni(II).

## 1 Introduction

Water pollution, especially heavy metal pollution has become an environmental problem concerned by the public. Chemical industry, electroplating, printing and dyeing, pharmaceutical and metallurgical industries can generate lots of heavy metal wastewater, which brings seriously threat to human health. Ni(II) is a common heavy metal ion in polluted wastewater, which is highly toxic and carcinogenic. Excessive intake of Nickel can damage brain, spinal cord and human internal organs (Li et al., 2017a). Besides, heavy Ni(II) pollution in water and soil will destroy the ecosystem and directly cause the reduction of crops and aquatic products (Lan et al., 2019). Therefore, it is necessary to take measures to deal with the heavy metal pollution represented by Ni(II) pollution in wastewater. The removal of Ni(II) ion from water has been widely concerned by scholars in the world. At present, the treatment technology of Ni(II) ion from heavy metal wastewater can be divided into chemical method (Li et al., 2009; Fu and Wang, 2010; Dong et al., 2011), biological method (Wen et al., 2018; Yu and Jiang, 2019) and physicochemical method (Cai et al., 2018; Yan et al., 2019) according to the treatment principle. The physicochemical method is widely used by combining physical and chemical effects to reduce the concentration of heavy metal ions in wastewater, so as to meet the discharge standard (Ghasemi and Rohani, 2019). Besides, the physicochemical method can achieve the recovery and utilization of heavy metal ions from wastewater.

Hydrotalcite compounds were often used to treat contamination of heavy metal ion. Hydrotalcite compounds, also known as layered double hydroxide (LDH), are a new kind of layered functional materials with brucite structure. The general chemical formula of hydrotalcite compounds was ${\left[M_{1-x}^{2+}M_{x}^{3+}{\left(OH\right)}_{2}\right]}^{x+}\left(A_{x/n}^{n-}\right).yH_{2}O$ . Where M^2+^ and M^3+^ represented the bivalent and trivalent metal cations that comprise the laminates, and A^n−^ represented the guest anions between the main laminates. M^2+^ was a divalent metal cation such as Mg^2+^, Ca^2+^, Zn^2+^, Cu^2+^, and Ni^2+^. The M^3+^ was a trivalent metal cation such as Al^3+^ and Fe^3+^. A^n−^ was referred to CO_3_^2-^, PO_4_^3-^, SO_4_^2-^, Cl^−^, OH^−^, NO_3_^−^ and other inorganic and organic ions as well as some complex ions (Wang and O’Hare, 2012). All kinds of divalent and trivalent metals can be incorporated into the framework of LDH. In addition, a complete LDH structure can be obtained when the mole ratio of M^2+^/M^3+^ was between 2 and 4, that was, the x value was between 0.2 and 0.33. Huang et al. synthesized Mg_2_Al-TCAS-LDHs and Zn_2_Al-TCAS-LDHs composite materials arranging between different TCAS layers to study the adsorption performance of TCAS-LDHS composite materials for toxic heavy metal ions. The maximum adsorption capacity of two kinds of TCAS-LDHS composite materials for Pb^2+^ were 205 mg‧g^−1^ and 188.1 mg‧g^−1^, while, the maximum adsorption capacity of two kinds of TCAS-LDHS composite materials for Cu^2+^ were 125 mg‧g^−1^ and 103.8 mg‧g^−1^ (Huang et al., 2016). Roozbeh et al. prepared functionalized Ni_50_Co_50_LDHs/UIO-66-(Zr)-(COOH)_2_ nanocomposites and used to remove Hg^2+^ and Ni^2+^ from wastewater solution. It was found that the adsorption capacity of Hg^2+^ and Hg^2+^ was as high as 509.8 mg‧g^−1^ and 441.0 mg‧g^−1^ (Soltani et al., 2021). However, due to the greenness of the material and the complicated synthesis methods, the further exploration was needed.

Controlling the chemical composition of inorganic layer cations and interlayer space anions in LDH, the unique structure and functional properties can be precisely regulated. Due to its unique interlayer structure and interlayer anion exchangability, LDH has a good application in removing heavy metal nickel. The main function of heavy metal removal include M(OH)_X_, aggregation on the surface of LDHs through precipitation, isomer substitution, bonding with -OH on the surface of LDHs, and chelation with functional ligands on the plate (Li et al., 2022).

Considering heavy metals captured from a complex water environment, the selective adsorbent of FeMgAl extracted from layered double hydroxide (LDH) with different intercalated anions (CO_3_^2-^, NO_3_^−^, and MoS_4_^2-^), and adsorbed Ni^2+^ ions were compared. The result showed that an isothermal optimized maximum adsorption amount of Fe-MoS_4_ was 369.0 mg g^−1^ (Ali et al., 2019). The chloride and heavy metal binding capacity of the hydrotalc-like phases formed in the binder were examined (Yang et al., 2020). The results showed that the binder had a higher anti-chloride ion permeability than silicate cement and other alkali-activated slag paste. When the binder dosage was 10%, the solidification efficiency of the Pb^2+^, Cu^2+^, and Cr^3+^ ions in the binder was between 66% and 88%. This study provided a guidance for the production of green binders for industrial solid waste treatment. The above hydroslide has made some progress in Ni^2+^ adsorption, but its adsorption effect can not be shown, and the influence of Ca-Fe-LDH adsorption on Ni^2+^ has not been explored.

According to the strength of metal KOH > Ca(OH)_2_ > NaOH > Mg(OH)_2_, LDH has a strong alkalinity, but the apparent alkalinity is small. The acidity of LDH is related to the alkalinity of the divalent metal hydroxide, the acidity of the trivalent metal hydroxide and the interlayer anion. The basic strength is consistent with that of divalent metal hydroxide, so Ca^2+^ is selected. In addition, due to the structure and chemical properties of Ca^2+^, Fe^3+^, Fe^2+^, Co^2+^ and Ni^2+^, isomorphic substitution is easy to occur (Liang et al., 2013). However, due to a series of problems such as secondary pollution caused by the application of Co^2+^ and harsh experimental conditions of Fe^2+^, Ca^2+^ and Fe^3+^ are finally determined to construct the unique interlayer structure of hydrotalcite.

Therefore, this study mainly focused on the preparation and characterization of Ca-Fe bimetallic hydroxide composite materials, and the preparation of layered double hydroxide composite materials was carried out by co-precipitation method to improve the selectivity and removal efficiency of nickel ion. Recovery experiments of nickel ion was conducted to explore the mechanism of adsorption of Ni^2+^ ion by LDH and the removal model of Ni^2+^ ion was established.

## 2 Materials and methods

### 2.1 Preparation of Ca-Fe-LDHs composite materials

CaCl_2_ and FeCl_3_.6H_2_O were purchased from Ptsrti. Anhydrous NaOH microspheres were provided with Macklin Corporation. All chemicals were 99% pure and did not require further purification.

Ca-Fe-LDHs was synthesized by coprecipitation method. Dissolving 90 mmol CaCl_2_ and 30 mmol FeCl_3_‧6H_2_O (Ca(II):Fe(III) = 3:1) into 100 ml water. The mixed salt solution was slowly added into the alkaline solution to keep the pH within the range of 12–13, and then violently stirring for 30 min. Then the suspension was stirred for 24 h at 25°C. The sediment was collected through filtration and washed three times with deionized water. Finally, LDHs samples were obtained after drying at room temperature.

### 2.2 Adsorption experiment of Ni^2+^ ion

NiSO_4_‧6H_2_O solution was used to simulate the wastewater containing Ni^2+^ ions. Weighing 4.4786 g NiSO_4_‧6H_2_O and dissolving the reagent in 1000 ml deionized water, so that the concentration of Ni^2+^ ions in the solution was 1.00 g/L. Hydrotalcite was put into nickel ion solution with the concentration of 1.00 g/L, and the change of concentration of Ni^2+^ ion was recorded during the 3 h reaction. The concentration of Ni^2+^ ions in solution was measured by inductively coupled plasma (ICP). All removal experiments were carried out in a magnetic stirrer with an initial pH of 7.0 and a temperature of 25°C. After the removal of Ni^2+^ ions, the solid samples were obtained with a 12.5 cm filter membrane and dried at 70°C. The separated liquid samples were collected for further characterization. All experiments were repeated with a reproducibility of ±5%. The removal capacity of Ca-Fe-LDHs for Ni^2+^ ions was defined as the intake of nickel ions per gram of original Ca-Fe-LDHs.

### 2.3 Characterization

The structure of prepared Ca-Fe-LDHs composite materials was analyzed by scanning electron microscope (SEM) (ZEISS Gemini 300). The crystal structure and composition of the prepared Ca-Fe-LDHs composite materials were analyzed with an X-ray powder diffractometer (XRD) (X’Pert Pro MPD). The test angle was 5–80^o^ and the test speed was 5 ^o^/min. Fourier transform infrared spectrometry (FTIR) (Thermo Scientific Nicolet iS20) was performed using a Nicolet iS50 spectrometer (Thermo Scientific) and the scanning wave was ranging from 400 to 4000 cm^−1^. The element valence of the Ca-Fe-LDHs composite material was determined by x-ray photoelectron spectroscopy (XPS) (Thermo Scientific K-Alpha). After taking appropriate powder sample tablet, put the sample into XPS instrument sample chamber. When the pressure of sample chamber is less than 2.0 × 10^−7^ mbar, send the sample to the analysis room, emit Al K ray (hv = 1486.6 eV) through X-ray source; spot size is 400 m, working voltage 12 kV, filament current 6 mA; full spectrum scan pass energy is 150 eV, step 1 eV; narrow spectrum scan pass energy is 50 eV, step 0.1 eV. The narrow spectrum performs at least 5 cycle signal accumulation (different element scanning times vary) for the binding energy correction: with the C1s = 284.80 eV binding energy as the energy standard. Data format is: VGD format and Excel format, where Excel format files are made with origin software drawing, VGD format files can be opened with Avantage analysis software.

## 3 Results and discussion

### 3.1 Characterization of Ca-Fe-LDHs composite materials

#### 3.1.1 FTIR analysis

**FIGURE 1:**
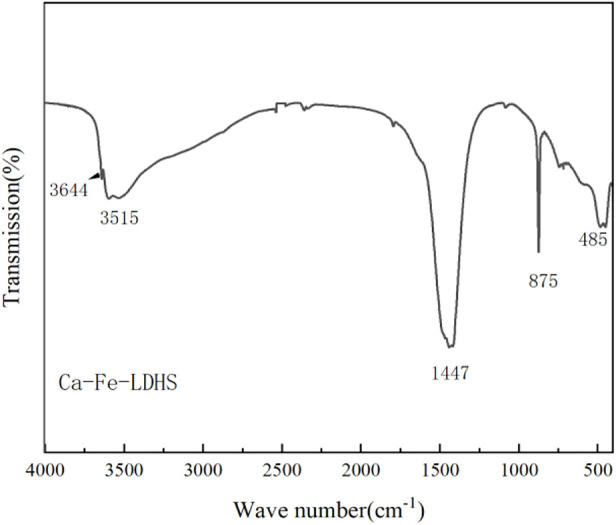
FTIR spectra of Ca-Fe-LDHs.

The FTIR spectrum of Ca-Fe-LDHs composite materials was shown in Figure 1. The band near 3644 cm^−1^ was due to the stretching vibration of -OH in Ca(OH)_2_. In the Ca-Fe-LDHS spectrum, the strong overlap band at 3515 cm^−1^ was attributed to the tensile vibration of the structural hydroxyl group (O-H) between the Ca-Fe-LDHs interlayers and lattice water. The deformation of Cl^−^ in Ca-Fe-Cl interlayer was about 989 cm^−1^. The peak at 493 cm^−1^ was attributed to the stretching vibration of M-O (M: Ca or Fe) and Ca-Fe-O in the lattice (Ifebajo et al., 2019). Moreover, due to the influence of air in the synthesis, the sandwich contained anionic CO_3_^2−^ (Frost et al., 2009), CO_3_^2−^ had two split bands around 1446 cm^−1^ (Wu et al., 2012). Therefore, according to the FITR spectrum, the synthesized material contained obvious structural hydroxyl and lattice water for structural support, and elements of Ca and Fe were participate in its tensile vibration process.

#### 3.1.2 XRD analysis

**FIGURE 2:**
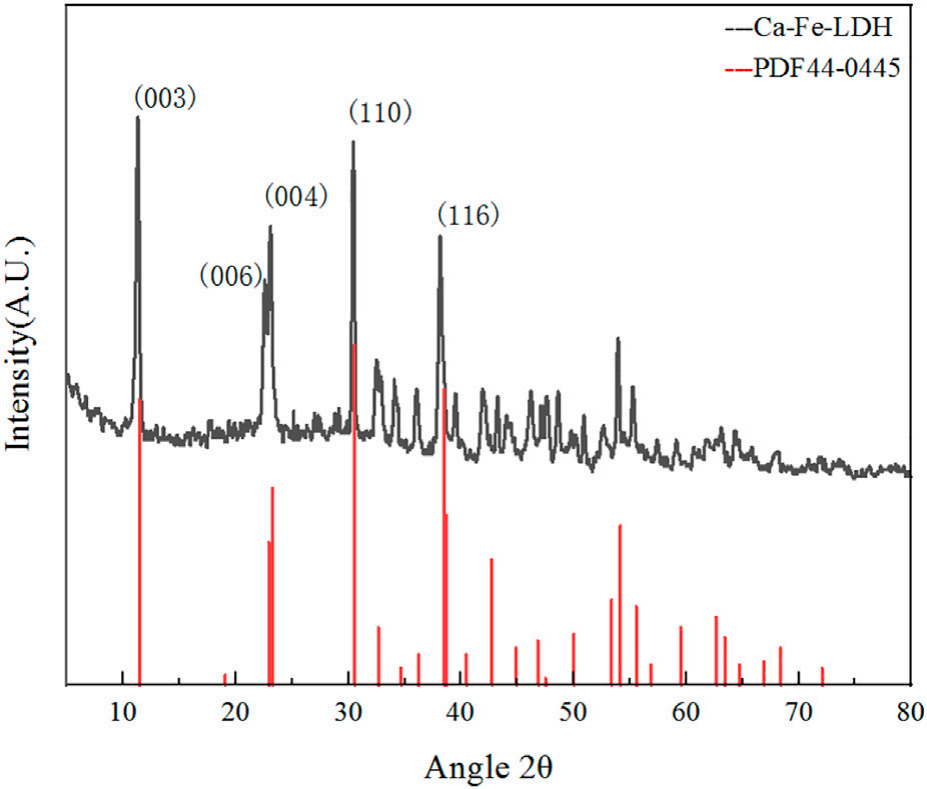
XRD spectra of Ca-Fe-LDHs.

XRD analysis of Ca-Fe-LDHs composite materials was illustrated in Figure 2. According to the standard card of PDF#44-0445, it showed that it was a layered structure material similar to Ca_2_Al(OH)_6_Cl(H_2_O)_2_·mH_2_O (Wu et al., 2009). Typical peaks were ascribed to (0 0 3), (0 0 6) and (1 1 0), which were consistent with PDF#44-0445 (Szabados et al., 2018). The lattice spacing of (0 0 3) plane was 0.788 nm, the lattice constant of a was 1.365 nm, and the average particle size d was 0.836 nm, which was close to the value of reported Ca_2_Al(OH)_6_Cl(H_2_O)_2_·mH_2_O. Compared with Mg-Al-LDH, a and d were larger due to the 7-coordination structure of Ca^2+^ (Millange et al., 2000). Therefore, it was indicated that the layered structure of the Ca-Fe-LDHs materials was generated, and metals of Ca and Fe were embedded reasonably. According to the results of high strength and wide line shape of (003) and (110), it indicated that hydrotalcite with relatively high crystallinity and smaller crystals.

#### 3.1.3 SEM analysis

**FIGURE 3:**
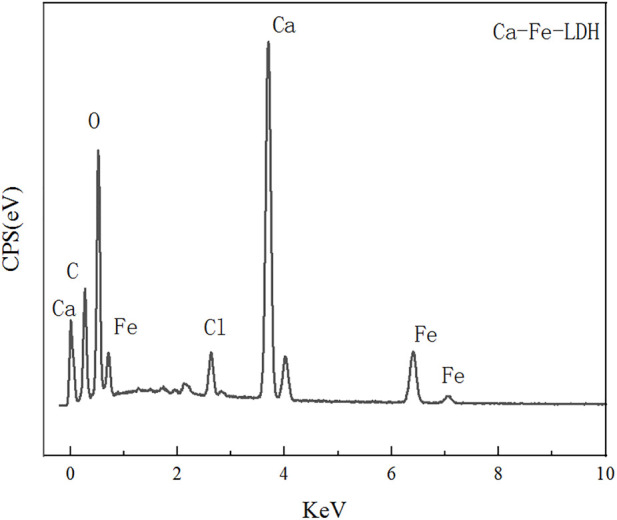
Energy spectrum of Ca-Fe-LDHs.

**FIGURE 4:**
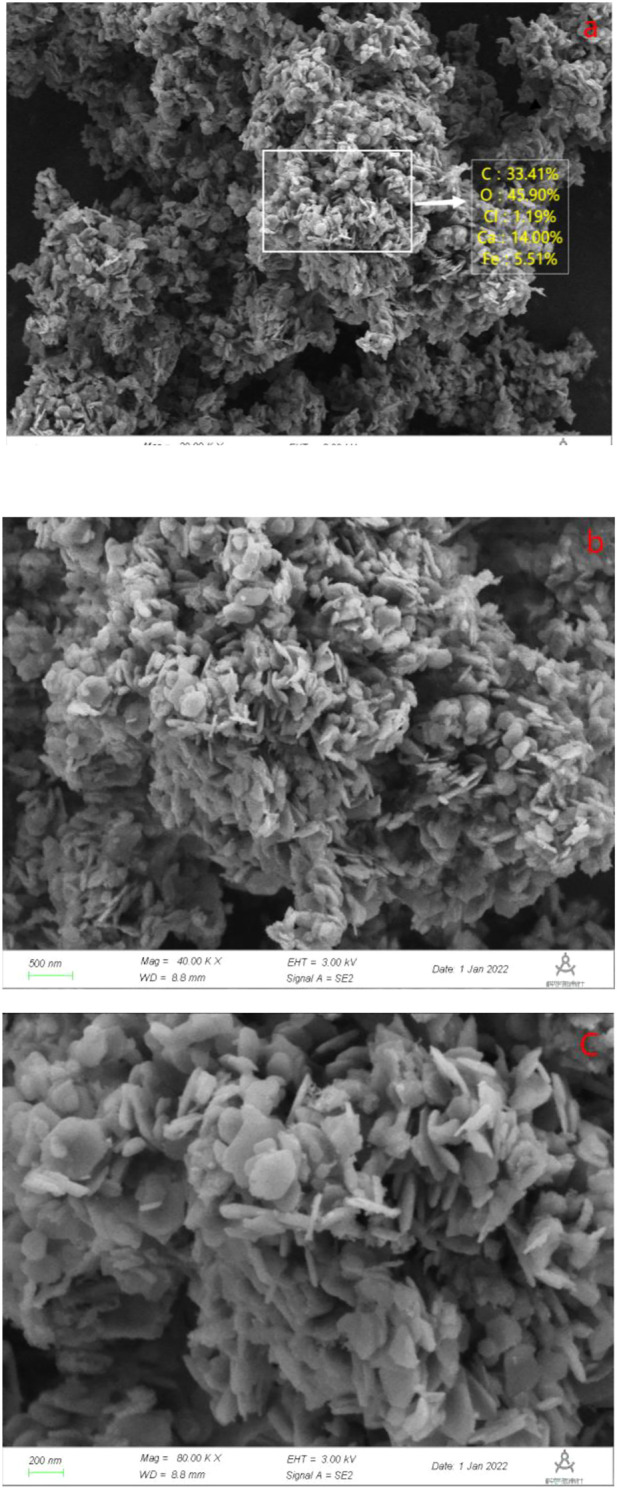
**(A)** SEM and element content diagram (Ca-Fe-LDHs, 20.00 KX), **(B)** SEM diagram (Ca-Fe-LDHs, 40.00 KX), and **(C)** SEM diagram (Ca-Fe-LDHs, 80.00 KX).

The morphology and composition of Ca-Fe-LDHs composite materials was analyzed by SEM. The energy spectrum of Ca-Fe-LDHs composite materials was shown in Figure 3. According to the results of Figure 3 and Figure 4, the atomic percentage of Ca/Fe was 14:5.51–2.5:1, and also contained Cl^−^. The result indicated that there was Cl^−^ in the interior of layered double hydroxide, but the contents of C and O were very high, indicating that the interior also contained CO_3_^2-^ ions. As shown in Figures 4B, C, the layered structures could be observed clearly. Therefore, it was shown that the Ca-Fe-LDHs material was successfully synthesized and its functionality was complete. The layered structure of Ca and Fe in the material was constructed in a reasonable proportion, and the atomic weight of O was the highest. Compared with the characterization of XRD and FTIR, -OH and lattice water were finally generated. However, the C existing in the Ca-Fe-LDHs composite materials indicated that the CO_2_ in the air showing an impact on the synthesis of product.

#### 3.1.4 XPS analysis

**FIGURE 5:**
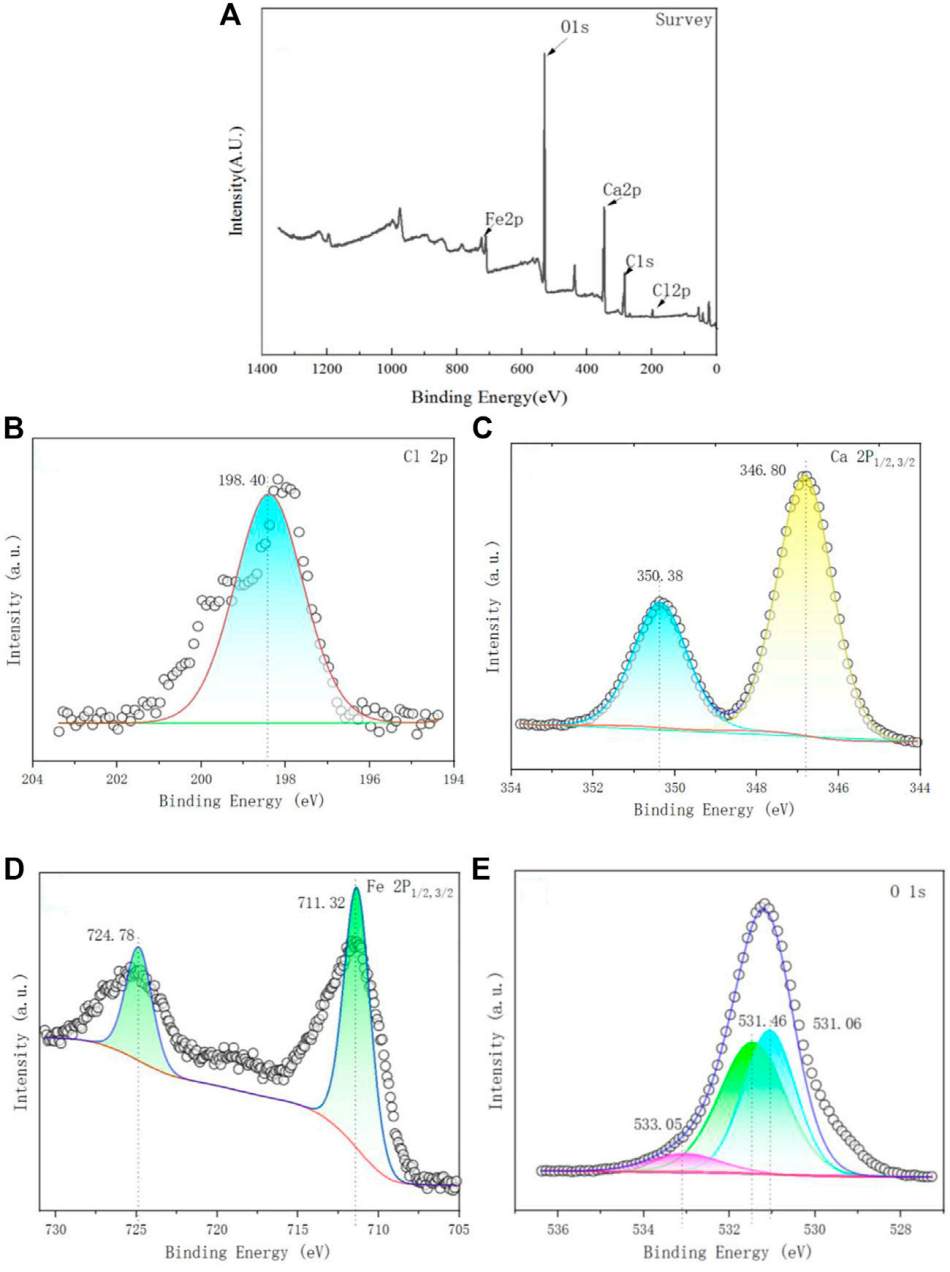
**(A)** XPS spectra of the Ca-Fe-LDHs; **(B)** High-resolution Cl; **(C)** High-resolution Ca; **(D)** High-resolution Fe; and **(E)** High-resolution O.

The composition of the samples and the oxidation state of the elements were determined by XPS. The measurements of the full spectral scan were shown in Figure 5A, observing key elements like Ca, Fe, Cl and O in the Ca-Fe-LDHs composite. As shown in Figure 5B, Cl 2p showed a peak at 198.4 eV, demonstrating the presence of Cl^−^. As shown in Figure 5C, the Ca 2p_3/2_ and Ca 2p_1/2_ peaks of the Ca-Fe-LDHs were located at 346.8 eV and 350.5 eV, respectively, reflecting the oxidation state of Ca^2+^. As shown in Figure 5D, the peaks of Fe 2p_3/2_ and Fe 2p_1/2_ were located at 711.4 eV and 724 eV, respectively, indicating the oxidation state of Fe^3+^. In addition, the area of the Ca: Fe was shown in Table 1, the area of the Ca: Fe was about 2:1, which was consistent with the results of the SEM analysis. As shown in Figure 5E, the 533.05 eV, 531.46 eV, 531.06 eV for O1s was the peaks of lattice oxygen (Fe-O/Ca-O), surface hydroxyl (OH), adsorption oxygen (Fe-O/Ca-O), which indicated the morphological changes of oxygen existence, and the adsorption oxygen and hydroxyl content were the most, which showed a huge impact with the subsequent adsorption. Thus, the results indicated that the presence of Ca or Fe ions on the surface of Ca-Fe-LDHs, which also confirmed the successful synthesis of Ca-Fe-LDHs.

**TABLE 1:** Basic information table of XPS before adsorption.

Peak	Pos. (eV)	Chemical stat	Area	%Area
Cl 2p	198.4	Cl^−^	8133.15	—
Ca 2p_1/2_	350.38	Ca-O	151734.1	—
Ca 2p3_/2_	346.8			
Fe 2p_1/2_	724.78	Fe-O	88132.07	—
Fe 2p_3/2_	711.32			
O 1s	533.05	(Fe-O/Ca-O) lattice oxygen	16226.02	6.96%
O 1s	531.46	-OH	114029.45	49.75%
O 1s	531.06	(Fe-O/Ca-O) adsorbed oxygen	98933.26	43.28%

### 3.2 Process of Ni(II) removing

#### 3.2.1 Adsorption performance of Ni(II)

**FIGURE 6:**
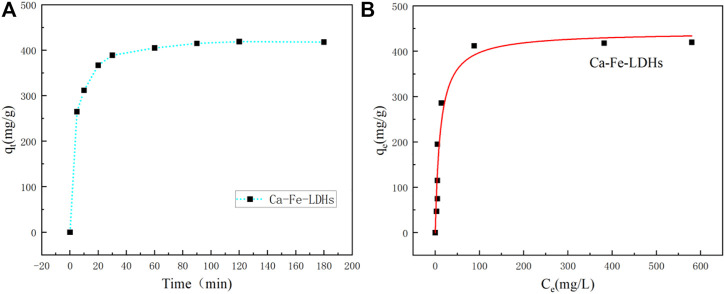
**(A)** Adsortime of Ni^2+^ ions by Ca-Fe-LDHs samples, and **(B)** Adsorption isotherms of the Ca-Fe-LDHs samples. (The q_t_ (mg/g) is the adsorption amount at time t; the q_e_ (mg/g) is the equilibrium adsorption amount).

As shown in Figure 6, it showed the effect of reaction time on the removal efficiency of Ni (II) for Ca-Fe-LDHs. Figure 6A showed that the adsorption amount of Ni (II) for Ca-Fe-LDHs was increased rapidly within 1 h and reached equilibrium after 3 h. The results showed that the adsorbent had a strong adsorption capacity for the metal ions within the first 1 h, and slowly reached the saturation state after 3 h. Figure 6B showed the adsorbed isotherm of Ca-Fe-LDHs samples obtained with Ni^2+^ concentrations of 50, 80, 120, 200, 300, 500, 800, and 1000 mg/L. The result showed a maximum adsorption amount of 418.9 mg g^−1^.

In order to better illustrate the adsorption performance, the adsorption isotherm analysis of Ni(II) for Ca-Fe-LDHs was carried out. In order to optimize the design of adsorption systems for Ni(II) ions removing from aqueous solutions, it was important to explain the relationship between the amount of Ni(II) ions adsorbed by per unit weight of adsorbent (*q*_*e*_) at adsorption equilibrium and the residual concentration of Ni(II) ions in solution. A large number of empirical models were used to analyze experimental data and describe how adsorbents and adsorbents interact. For example, Langmuir, Freundlich, Temkin, Dubinin-Radushkevich and other isotherm models were used to explain the results of adsorption studies.

The corresponding equations were given:

$${Langmuir:q}_{e}=\frac{q_{m}K_{L}C_{e}}{1+K_{L}C_{e}}$$ (1)

$${Freundlich:q}_{e}{=K}_{F}{C_{e}}^{1/n}$$ (2)

where q_m_ (mg/g) and K_L_ (L/mg) are Langmuir isotherm coeffifi-cients; K_F_ (mg/g) and n are Freundlich constants; C_e_ (mg/L) is the adsorption equilibrium concentration.

**FIGURE 7:**
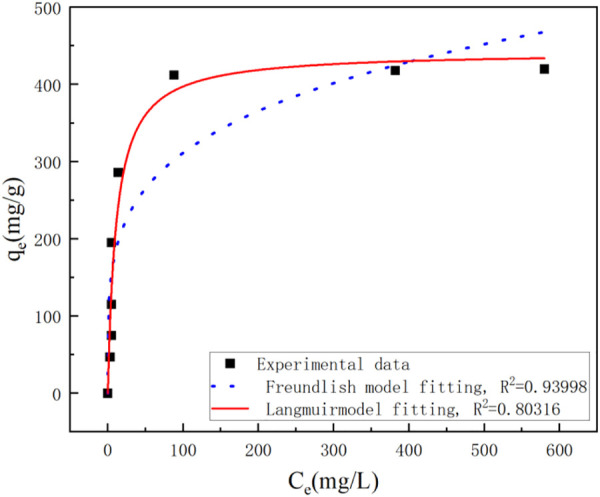
Analysis of adsorption isotherm.

Figure 7 showed the Langmuir and Freundlich (adsorption isotherms) applied to our experimental data. Table 2 showed the different parameters of all adsorption isotherms for Ni (II) ion removal using Ca-Fe-LDHs as adsorbent. According to Langmuir adsorption isotherm, the maximum adsorption capacity (q_max_) of Ca-Fe-LDHs was up to 442.561 mg g^−1^. The K_L_ value of Langmuir adsorption strength was 11.376 L mg^−1^. According to the Freundlich adsorption isotherm, the value of nF indicated the advantage of adsorption. The results showed that the nF value of Ca-Fe-LDHs adsorbed Ni (II) ions was 3.57, indicating that the Langmuir isotherm was a monolayer and had a certain adsorption effect. In addition, it was concluded that all adsorption sites on Ca-Fe-LDHs had the same energy. In the fitting results, Langmuir adsorption isotherm model was better than Freundlich adsorption isotherm model fitting the experimental data of Ca-Fe-LDHs adsorption of nickel ions. The results showed that there were surface active sites on Ca-Fe-LDHs for Ni(II) adsorption. As shown in Table 3, compared with the maximum adsorption capacity of some reported adsorbents, Ca-Fe-LDHs also showed an excellent adsorption capacity for Ni(II).

**TABLE 2:** Adsorption isotherms.

Absorbent	T(K)	Langmuir			Freundlich		
		Qmax	K_L_	*R* ^2^	K_F_ (mg^1-1/n^L^1/n^g^−1^)	n	*R* ^2^
		(mg.g^−1^)					
Ca-Fe-LDHs	298.15	442.5609 ± 27.79351	11.3761 ± 2.69325	0.93998	107.06683 ± 30.98092	0.23182 ± 0.05425	0.80136

**TABLE 3:** Comparison of Ni(II) adsorption capacity of related adsorbents in literature.

Adsorbent	Variety	Q_e_ (mg.g^−1^)	References
Penicillium	biomass	63.6	Sundararaju et al. (2020)
Findustrial waste brewery sludge	biomass	7.874	Kulkarni Rajeswari et al. (2019)
PVP-modified aluminosilicates	powder	17.023	Pomazkina et al. (2018)
Glycine functionalized graphene oxide (GO-G)	powder	90.90	Najafi et al. (2015)
Thermally modifed diatomite	powder	45.96	Farzane and Delavari, (2022)
A magnetic new nanocomposite	powder	8.63	Kucukcongar et al. (2020)
Soil components	biomass	0.325	Zahedifar and Moosavi, (2020)
Sewage sludge bio char (SBC)	powder	35.50	Yang et al. (2019)

### 3.3 Characteristics of Ca-Fe-LDHs after Ni(II) ions adsorption

#### 3.3.1 FTIR analysis of Ca-Fe-LDHs after Ni(II) ions adsorption

**FIGURE 8:**
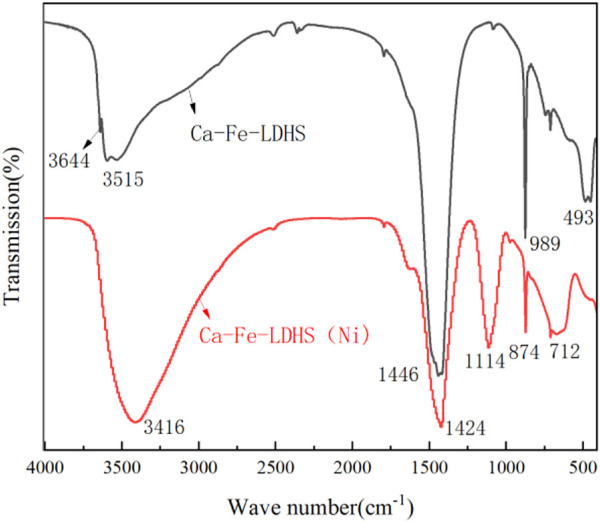
FTIR spectra of Ca-Fe-LDHs after Ni^2+^ ion adsorption.

Figure 8 showed the FTIR spectra of Ca-Fe-LDHs after Ni(II) ions adsorption. The scanning test range was from 450 cm^−1^ to 4,000 cm^−1^. The FTIR spectra showed a dissolution of LDH, as the bands of H-O-H decreased at 3,500–3,650 cm^−1^. It showed that a peak at about 3,416 cm^−1^ was attributed to the broad band vibration of hydrolyzed H-CaF-LDH hydroxylation (−OH), and the peak at about 1,114 cm^−1^ was the broad shoulder band spectrum of SO_4_^2−^, which proved that SO_4_^2−^ was absorbed in the simulated wastewater produced with Ni_2_SO_4_ as raw material. The Cl^−^ characteristic spectrum at 874 cm^−1^ was weakened, indicating that SO_4_^2−^ or CO_3_^2−^ had been replaced. The oscillating band at 712 cm^−1^ was a ferric hydroxide structure, which was the main component of the residual solid after the release of some Ca^2+^during the hydrolysis of LDH, indicating that the dissolution of LDH led to the formation of calcium-containing or ferric nickel hydroxide (Ni/Ca-FeOH). However, there was a strong peak at 1424 cm^−1^, which proved that CO_3_^2-^ existed in the adsorbed material.

#### 3.3.2 XRD analysis of Ca-Fe-LDHs after Ni(II) ions adsorption

**FIGURE 9:**
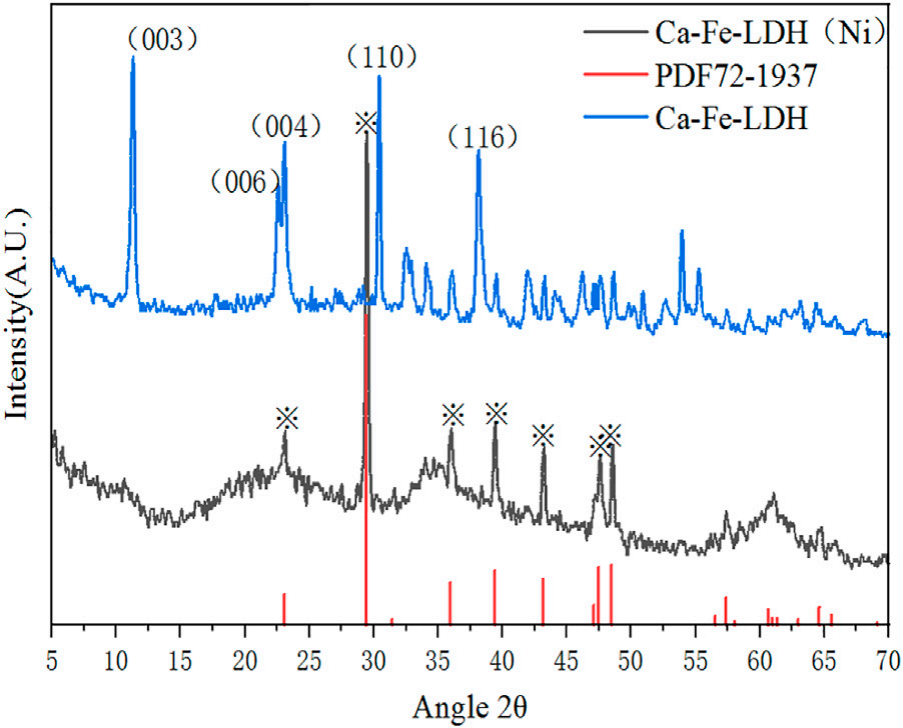
XRD spectra of Ca-Fe-LDHs after Ni^2+^ ion adsorption.

Figure 9 showed the spectrum of Ca-Fe-LDHs after Ni(II) ions adsorption. Due to the poor stability of Ca-Fe-LDHs in aqueous solution, only calcium carbonate was found in the collected solids (Ca-Fe-LDHs). Therefore, during the adsorption of heavy metals, as time increasing, Ca-Fe-LDHs were gradually hydrolyzed, and the crystallization state was damaged (Sipiczki et al., 2013).

#### 3.3.3 SEM analysis of Ca-Fe-LDHs after Ni(II) ions adsorption

**FIGURE 10:**
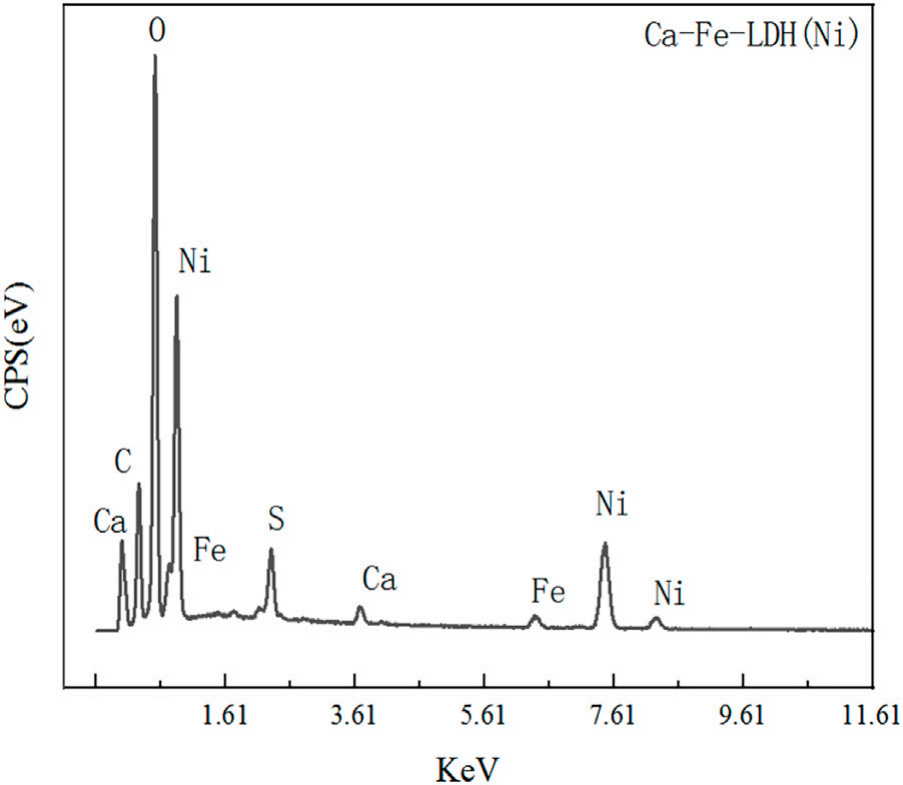
Energy spectrum of Ca-Fe-LDHs after Ni^2+^ ion adsorption.

**FIGURE 11:**
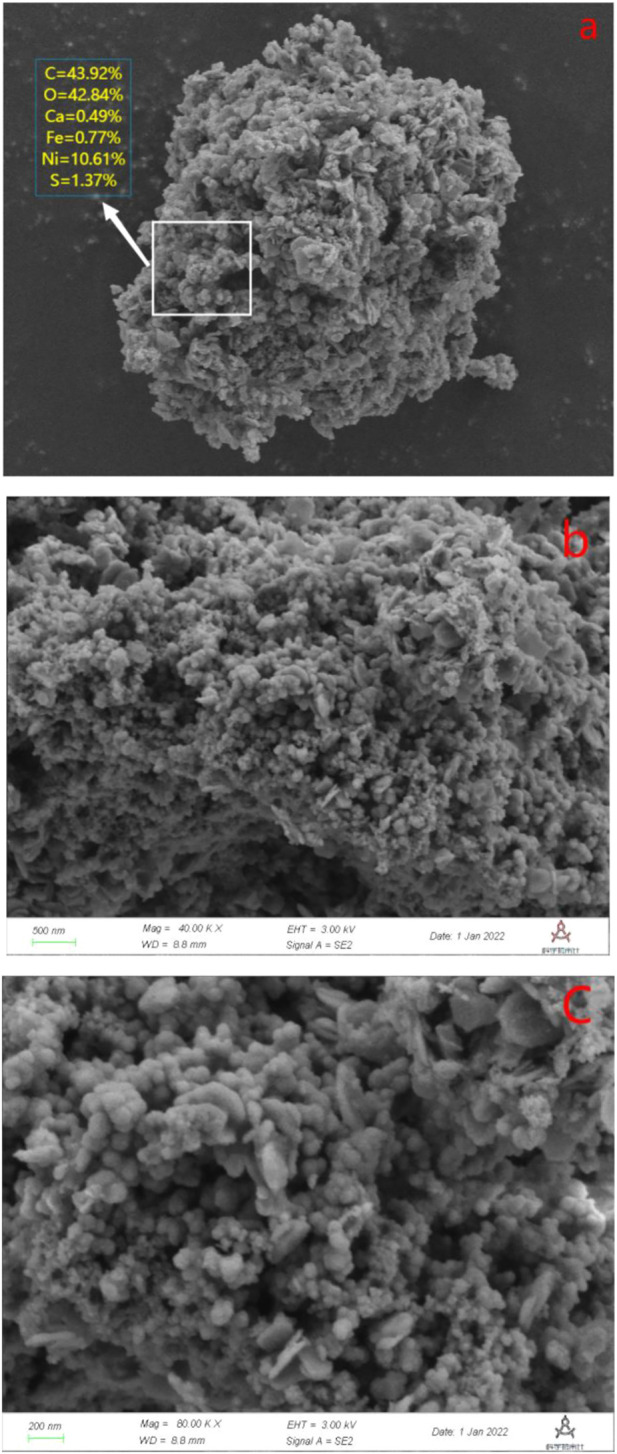
**(A)** SEM and element content diagram for Ca-Fe-LDHs after Ni^2+^ ion adsorption (Ca-Fe-LDHs after Ni^2+^ ion, 20.00 KX); **(B)** SEM diagram (Ca-Fe-LDHs after Ni^2+^ ion adsorption, 40.00 KX); and **(C)** SEM diagram (Ca-Fe-LDHs after Ni^2+^ ion adsorption, 80.00 KX).

Energy spectrum scanning was carried out and shown in Figure 10, the rate of Ca/Fe was 0.63. Besides, the content of Ni(II) was 10.61% and the rate of Ni/Fe was 13.85, which was much higher than the content of Fe^3+^ and Ca^2+^, indicating that the surface adsorption was greater than the ion exchange, which was consistent with the previous result. SEM analysis was carried out on the Ca-Fe-LDHs material after the adsorption of Ni(II) ions by Ca-Fe-LDHs and shown in Figure 11. It could be clearly seen that the outer layer structure of the material was wrapped by some substances in the state of microspheres, which was attributed to the adsorption of Ni(II) ions. The S element was observed in the material, indicating that SO_4_^2−^ ion was also absorbed by the material or ion exchange. It can be explained that most of the removal of Ni(II) by Ca-Fe-LDHs was attributed to the surface adsorption, and the role of ion exchange was very small. Lastly, synthesis conditions of ion exchange was not met according to literature review (Daud et al., 2019).

#### 3.3.4 XPS analysis of Ca-Fe-LDHs after Ni(II) ions adsorption

**FIGURE 12:**
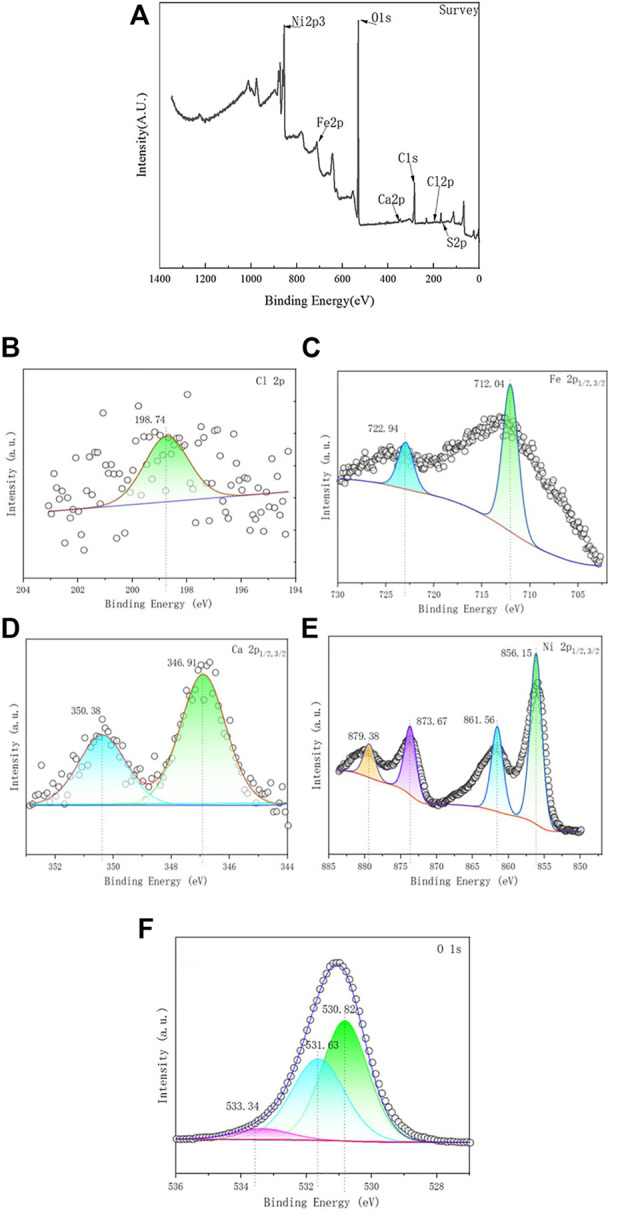
**(A)** XPS spectra of the Ca-Fe-LDHs after Ni^2+^ ion adsorption; **(B)** High-resolution Cl; **(C)** high-resolution Fe; **(D)** high-resolution Ca; **(E)** high-resolution Ni; and **(F)** high-resolution O.

In order to determine the ability of the Ca-Fe-LDHs to absorb the Ni (II) ions, we further characterized the samples using XPS. Basic information of XPS for Ca-Fe-LDHs after adsorption was shown in Table 4. As shown in Figure 12A, the key elements such as Ca, Ni, Fe, Cl, O and S were observed, indicating that the SO_4_^2−^ was absorbed. From the mass content analysis, the area ratio of Ni/Fe/Ca was about 100:4:1. According to XRD, the hydrolysis effect was relatively obvious, so the Fe content was less and the sharp decrease of Ca indicated that Ni(II) replacing Ca(II), resulting in a sharp decrease in the content. As shown in Figure 12B, Cl 2p showed a peak at 198.7 eV, demonstrating the presence of Cl^−^. As shown in Figure 12C, the peaks of Fe 2p_3/2_ and Fe 2p_1/2_ were located at 713.3 eV and 724 eV, respectively, indicating the oxidation state of Fe^3+^. Similarly, as shown in Figure 12D, the peaks of Ca 2p_3/2_ and Ca 2p_1/2_ were located at 346.8 eV and 350.5 eV, respectively, reflecting the presence of Ca^2+^ oxidation state, and the main adsorption was that Fe-O interacted with the surface hydroxyl group. As shown in Figure 12E, the peaks of Ni 2P_3/2_, Ni 2P_3/2_, Ni 2P_1/2_, and Ni 2P_1/2_ were located in 856.2 eV, 861.6 eV, 873.5 eV and 879.9 eV, respectively, indicating the generation of Ni(OH)_2_, Ni-O, NiOOH, etc. The formation of Ni (II) indicated the presence of various types of Ni (II) on the surface, which was closely related to the distribution of adsorbed oxygen and the -OH structure in the hydrotalc form. As shown in Figure 12F, the 533.34 eV, 531.63 eV, 530.82 eV in O1s were the peaks of lattice oxygen (Ni-O), surface hydroxyl (OH) and adsorbed oxygen (Ni-O), and the hydroxyl group was more abundant than the adsorption oxygen content, indicating that most of them were combined with the hydroxyl group to produce chemical adsorption. The formation of the lattice oxygen (Ni-O) indicated that a small amount of the Ni had synthesized the new Ni-Fe-LDHs.

**TABLE 4:** Basic information table of XPS after adsorption.

Peak	Pos. (eV)	Chemical stat	Area	%Area
Cl 2p	198.4	Cl-	850.74	—
Ca 2p_1/2_	350.38	Ca-O	5686.24	—
Ca 2p_3/2_	346.8			
Fe 2p_1/2_	724.78	Fe-O	28678.87	—
Fe 2p_3/2_	711.32			
O 1s	533.34	(Fe-O/Ca-O) lattice oxygen	12113.81	5.35%
O 1s	531.63	-OH	93320.23	41.18%
O 1s	530.82	(Fe-O/Ca-O) adsorbed oxygen	120489.68	53.48%
Ni 2p_1/2sat_	879.38	Ni-O	25736.15	9.52%
Ni 2p_1/2_	873.67	Ni(OH)_2_	52515.25	19.05%
Ni 2p_3/2sat_	861.56	NiOOH	64993.96	23.81%
Ni 2p_3/2sat_	856.15	Ni(OH)_2_	130596.64	47.62%

### 3.5 Mechanism of Ni(II) wastewater treatment using Ca-Fe-LDHs materials

LDHs showed a good adsorption performance on heavy metal ions due to the ability of exchange and adsorption for ions between layers, so it was used to develop materials for adsorption of various heavy metal ions. In general, for common heavy metal cations, LDHs removed heavy metal cations through the following mechanisms, including the formation of surface sediments, such as oxides, hydroxides, carbonates. Ions adsorption was achieved by surface hydroxyl bonding with LDH, isocrystalline replacement, and bonding with functional ligands in the sandwich medium. In addition, cation exchange (isocrystalline replacement) as a non-specific adsorption, was difficult to occur during low concentration adsorption (Li et al., 2017b; Yu et al., 2018).

**FIGURE 13:**
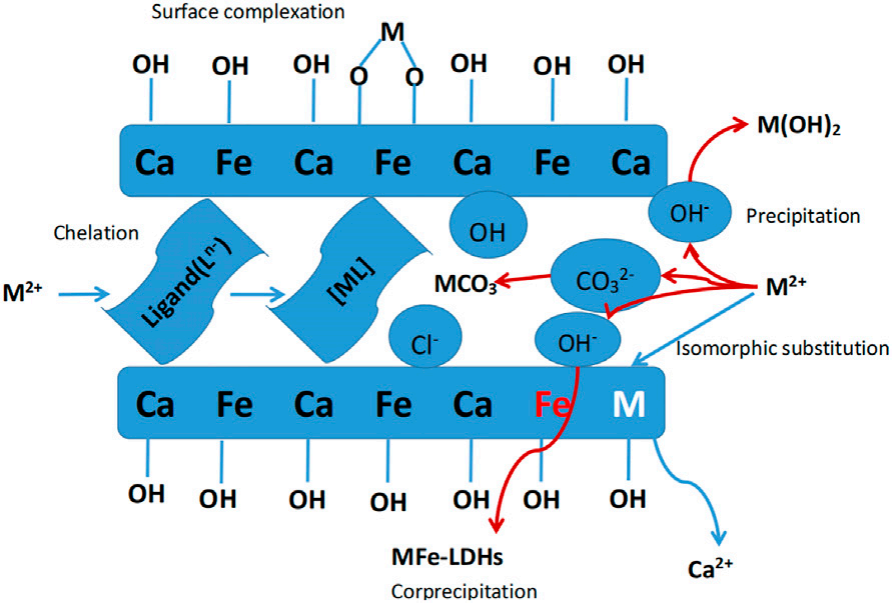
Diagram of Ni^2+^ ion adsorption process using Ca-Fe-LDHs.

In the adsorption experiment, large number of hydroxyl groups on the surface of Ca-Fe-LDHs provided a high adsorption capacity for Ni(II). At the same time, the intensity of hydroxyl peak decreased after adsorption, indicating that Ni(II) and its hydrolysate were involved in hydroxyl interaction on Ca-Fe-LDHs surface. In addition, according to FTIR spectra, Ca ions could undergo isomorphic mass exchange reaction and ion exchange with Ni(II) during the adsorption process (Kucukcongar et al., 2020). The removal mechanism of Ni(II) using Ca-Fe-LDHs was shown in Figure 13, Although the hydrolysis of Ca-Fe-LDHs occurred, its electrostatic interaction and hydrogen bonding might be the driving forces of the adsorption process due to hydrolysis. Moreover, it could also be seen from a series of results that the adsorption removal of Ni(II) mainly depended on isothermal mass exchanging and surface capturing. The results of XPS and SEM showed that the effect of surface capturing was much stronger than isothermal mass exchanging. This was because the synthesis of Ni-Fe-LDHs needed to be carried out in a certain alkaline environment. Because Ni(OH)_2_ precipitation would occur under alkaline conditions, and it was impossible to judge the adsorption effect of hydrotalcite, so the ion exchange precipitation was not ideal. However, the unique structure of hydrotalcite enabled its surface capturing to achieve a strong effect.

## 4 Conclusion

Ca-Fe-LDHs was successfully prepared by coprecipitation method and characterized by FTIR, XRD, SEM and XPS techniques. The results showed that the synthesized material had layered structures, and the content ratio and chemical composition of Ca and Fe elements were well presented. After the treatment of Ni(II) wastewater using Ca-Fe-LDHs materials, the maximum adsorption capacity of Ni(II) ions by Ca-Fe-LDHs was 418.9 mg‧g^−1^ within 3 h. The results showed that Ni(II) ions was more adsorbed on the surface of hydrotalcite and reacted with hydroxyl group. However, in the experiments of Ni(II) adsorption by Ca-Fe-LDHs, it was found that Ca-Fe-LDHs was hydrolyzed and only a small amount of Ni-Fe-LDHs structure was found. Therefore, isomorphic transformation and surface capture were attributed to the Ni(II) adsorption during the removal process of Ni(II) ions using Ca-Fe-LDHs, among which surface capture was the main adsorption mode.

## Data Availability

The raw data supporting the conclusion of this article will be made available by the authors, without undue reservation.
